# Molecular Cloning and Expression Analysis of the Cryptochrome Gene *CiPlant-CRY1* in Antarctic Ice Alga *Chlamydomonas* sp. ICE-L

**DOI:** 10.3390/plants11172213

**Published:** 2022-08-26

**Authors:** Yaoyao Zhao, Zhou Zheng, Xin Zhang, Yating Bao, Jinlai Miao

**Affiliations:** 1 Department of Specialty Medicine, School of Basic Medicine, Qingdao University, Qingdao 266071, China; 2 Key Laboratory of Marine Eco-Environmental Science and Technology, First Institute of Oceanography, Ministry of Natural Resource, Qingdao 266061, China; 3 Laboratory for Marine Drugs and Bioproducts, Qingdao National Laboratory for Marine Science and Technology, Qingdao 266237, China

**Keywords:** *CiPlant-CRY1*, *Chlamydomonas* sp. ICE-L, cryptochrome, extreme Antarctic environments, heterologous expression

## Abstract

Cryptochrome (CRY) is a kind of flavin-binding protein that can sense blue light and near-ultraviolet light, and participates in the light response of organisms and the regulation of the circadian clock. The complete open reading frame (ORF) of *CiPlant-CRY1* (GenBank ID OM389130.1), encoding one kind of CRY, was cloned from the Antarctic ice alga *Chlamydomonas* sp. ICE-L. The quantitative real-time PCR study showed that the expression level of the *CiPlant-CRY1* gene was the highest at 5 °C and salinity of 32‰. *CiPlant-CRY1* was positively regulated by blue or yellow light, suggesting that it is involved in the establishment of photomorphology. The *CiPlant-CRY1* gene can respond to polar day and polar night, indicating its expression is regulated by circadian rhythm. The expression level of *CiPlant-CRY1* was most affected by UVB irradiation, which may be related to the adaptation of ice algae to a strong ultraviolet radiation environment. Moreover, the recombinant protein of *CiPlant-CRY1* was expressed by prokaryotic expression. This study may be important for exploring the light-induced rhythm regulation of Antarctic ice algae in the polar marine environment.

## 1. Introduction

Light is an important environmental factor that affects life systems on Earth. It is not only an energy source for photosynthesis, but also a signal source for the growth and development of photosynthetic organisms. The light–dark cycle is one of the most regular periodic signals of organisms, controlling the metabolism of most species, rhythmic changes in physiology, and behavior. In addition to the beneficial effects of light, its ultraviolet rays can also cause DNA damage. As an important photoreceptor and transcriptional regulator, cryptochrome (CRY) is a kind of photochrome that affects plant growth and development, metabolism, and its own phototropism by absorbing blue light (400–500 nm) and near-ultraviolet light (320–400 nm). It is a photoreceptor that participates in the process of phototransduction and the formation of the biological clock in vivo [1]. The CRY-mediated light signaling pathway is essential for plant growth and development and adaptation to changes in the external environment, and the research on the mechanism of CRY action is a hot spot and frontier in the field of international plant physiology research.

In plants, CRYs are mainly involved in the regulation of photomorphogenesis, including the regulation of plant circadian rhythms, stomatal opening, root growth, osmotic stress response, shade avoidance, leaf senescence, and other circadian rhythms and life cycle processes [2]. CRY generally has two domains, including an amino-terminal photolyase homology region (PHR) that binds to the flavin chromophore, and a carboxy-terminal C-terminal extension responsible for signaling [3]. According to sequence similarity, plant CRY family genes can be divided into three subfamilies: plant CRY, animal CRY, and CRY-*Drosophila*, *Arabidopsis*, *Synechocystis*, *Homo* (CRY-DASH) [4,5]. The light signal transmission mediated by plant CRY photoreceptors mainly includes three molecular processes: blue light-induced photoreduction, conformational change to form active dimers, and binding to downstream proteins to transmit light signals [6]. Despite advances in characterization of CRYs, their photochemical, regulatory, and light-induced structural changes remain to be addressed.

At present, the research on marine algal CRY has just started, and there are few reports on the low-temperature algal CRY, and in-depth research is urgently needed. Light is the most influential driver of Antarctic ice algal communities. Blue light can penetrate to substantial depths in seawater. Blue light receptors play an important role in the adaptation of algae to the changing marine environment [7]. At present, the research on algal CRY mainly focuses on CryP and CPF1. CryP is involved in regulating the input pathway of the circadian clock, has an inhibitory effect on the reset of the circadian clock by blue light, and is also associated with circadian rhythm changes, affecting the accumulation of light-harvesting proteins [8]. *Volvox carteri* and *Chlamydomonas reinhardtii* each have two CRY-DASHs, but they have not been characterized to date [9]. Antarctic ice algae can thrive in the Antarctic polar day and night and sea ice environment with strong ultraviolet radiation, while room temperature marine algae cannot survive. Antarctic ice algae must have special physiological mechanisms to maintain their survival and reproduction [10]. Obviously, compared with normal temperature plant CRY, Antarctic ice alga CRY can regulate physiological functions in the sea ice environment with extreme light–dark cycles and strong ultraviolet radiation, and must have a special mechanism of action.

We have cloned the complete open reading frame (ORF) of *CiCRY-DASH1* (GenBank ID MK392361) from the Antarctic ice alga *Chlamydomonas* sp. ICE-L, and performed related characterizations [11]. The CRYs of *Chlamydomonas* sp. ICE-L may be related to how ice algae adapt to extreme Antarctic environments. Here, we study a novel plant-type CRY gene *CiPlant-CRY1* in *Chlamydomonas* sp. ICE-L, which may provide a new perspective for the adaptation of Antarctic ice alga CRY to the polar marine environment and its physiological characteristics.

## 2. Results

### 2.1. Bioinformatics Analysis of CiPlant-CRY1

**Figure 1:**
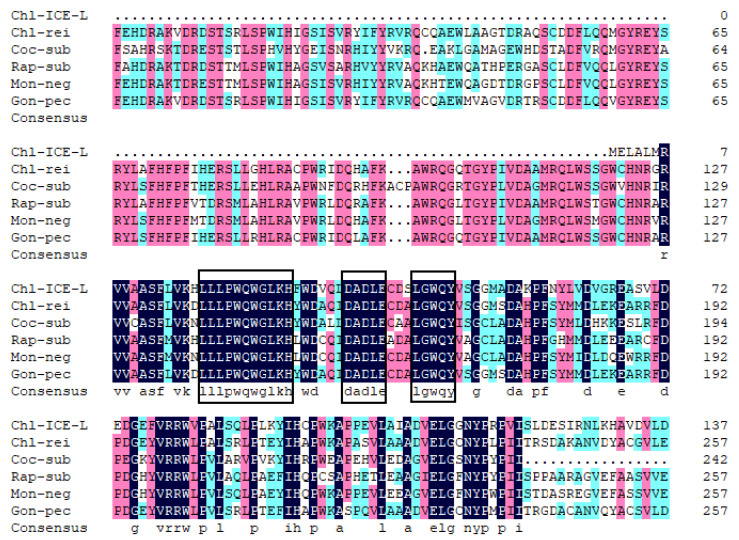
Alignment comparison of the deduced amino acid sequences of CiPlant-CRY1 with other species. Chl-ICE-L: *Chlamydomonas* sp. ICE-L, Chl-rei: *C. reinhardtii*, Coc-sub: *C. subellipsoidea* C-169, Rap-sub: *Raphidocelis subcapitata,* Mon-neg: *Monoraphidium neglectum*, Gon-pec: *Gonium pectoral*. The frame represents the conservative domain of Plant-CRY protein.

As shown in Figure 1, the amino acid sequence of *CiPlant-CRY1* contains the PHR domain, a protein conserved functional domain shared by CRY, while the C-terminal extension is not found.

Bioinformatics analysis showed that *CiPlant-CRY1* had 1452 base pairs, encoding 483 amino acids, and its molecular formula was C_2293_H_3498_N_648_O_729_S_16_ and relative molecular weight was 52.32 kDa, and as an acidic protein, its PI was 4.90. Its instability index (II) was calculated as 37.82, which indicated that the protein was stable. Its average coefficient of hydrophilicity GRAVY was −0.641, and its aliphatic index was 64.47. The secondary structure of *CiPlant-CRY1* showed that helix accounted for 20.29%, strand for 2.69%, and loop for 77.02%. Analysis of its transmembrane profile by TMPred showed that there may be no transmembrane proteins. In addition, the subcellular localization of the *CiPlant-CRY1* gene was predicted by the WoLF PSORT online tool, showing that *CiPlant-CRY1* is likely to be located in the nucleus.

**Figure 2:**
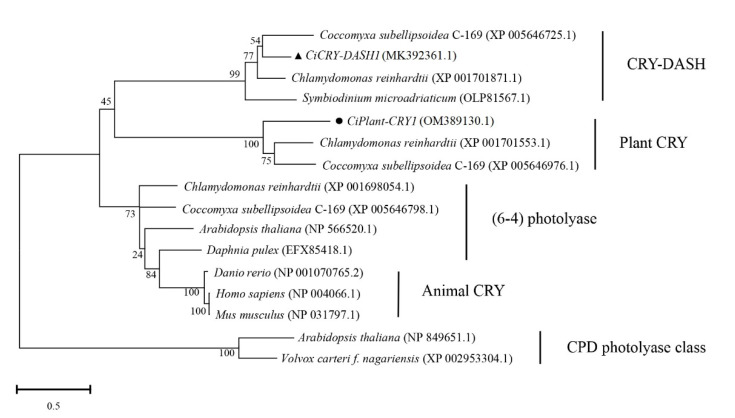
Phylogenetic tree for *CiPlant-CRY1* and related cryptochrome/photolyase family based on amino acid sequences. Numbers at nodes indicate levels of bootstrap support (%) based on a neighbor-joining analysis. The bootstrap test of the tree was performed with 1000 replications.

As shown in Figure 1, the conserved amino acid domains are drawn in three frames, and these conserved amino acid domains are located in the FAD binding site region. The amino acid sequence alignment analysis showed that CiPlant-CRY1 of *Chlamydomonas* sp. ICE-L had the highest homology (34.48%) with *C. reinhardtii*, indicating that the species had the closest affinity. It also had homology with *Gonium pectoral* (34.10%), *Monoraphidium neglectum* (32.57%), and *Raphidocelis subcapitata* (29.12%), and it had the lowest homology (27.97%) with *C. subellipsoidea* C-169. The phylogenetic tree (Figure 2) showed that *CiPlant-CRY1* belonged to plant type CRY, and was most closely related to the plant type CRY of *Chlamydomonas reinhardtii*.

### 2.2. Effects of Different Stress Conditions on the Expression of CiPlant-CRY1

**Figure 3:**
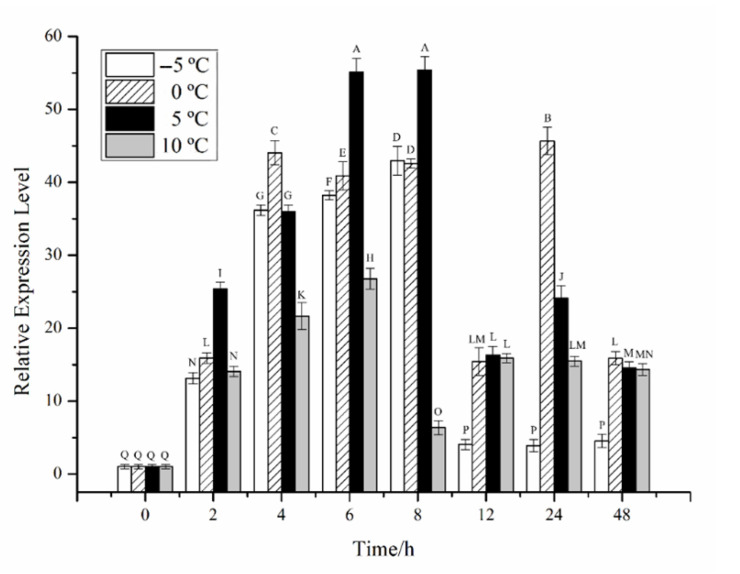
Relative expression of the *CiPlant-CRY1* at different temperatures for different times. Standard error bars are shown, n = 3. Different capital letters indicate significant differences between different treatments (*p* < 0.01).

The effects of temperature stress on *CiPlant-CRY1* at the transcriptional level are shown in Figure 3. The expression level of *CiPlant-CRY1* at −5 °C reached a maximum value of 42.612 at 8 h. The expression level of *CiPlant-CRY1* at 0 °C decreased after reaching 44.058 at 8 h and reached a maximum value of 45.653 at 24 h. At a normal temperature of 5 °C, the expression level of *CiPlant-CRY1* reached a maximum value of 55.403 at 8 h, which was the maximum expression level (*p* < 0.01). The expression level of *CiPlant-CRY1* at 10 °C reached a maximum value of 26.784 at 6 h, and its trend changes are small (*p* < 0.01).

**Figure 4:**
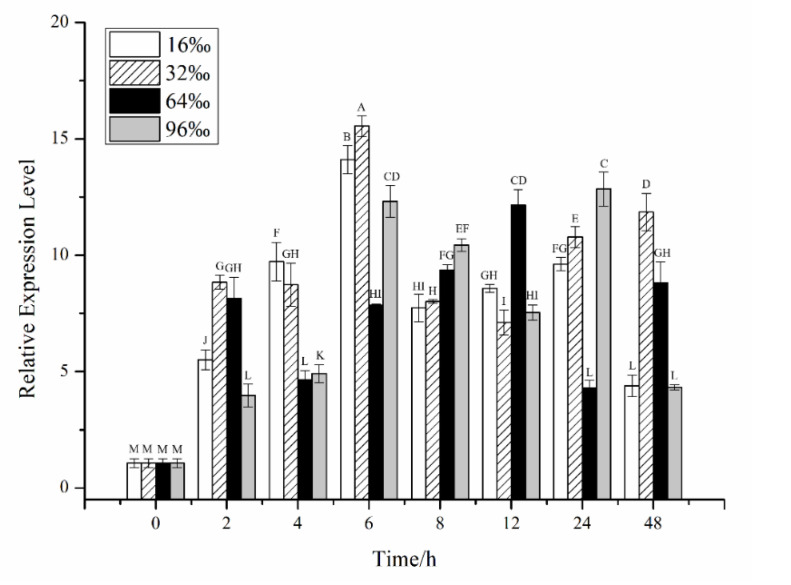
Relative expression of the *CiPlant-CRY1* in salinity of 16‰, 32‰, 64‰, and 96‰ for different times. Standard error bars are shown, n = 3. Different capital letters indicate significant differences between different treatments (*p* < 0.01).

The effects of salinity stress on *CiPlant-CRY1* at the transcriptional level are shown in Figure 4, and the expression levels of *CiPlant-CRY1* fluctuated. The expression level of *CiPlant-CRY1* at salinity of 16‰ reached the highest value of 14.111 at 6 h, but was still slightly lower than 32‰. At the normal salinity of 32‰, the expression level of *CiPlant-CRY1* reached the highest value of 15.549 at 6 h. At salinity of 64‰, the expression level of *CiPlant-CRY1* reached the highest value of 12.155 at 12 h (*p* < 0.01). The expression level of *CiPlant-CRY1* at salinity of 96‰ was higher than other experimental groups at 8 h and 24 h (*p* < 0.01).

**Figure 5:**
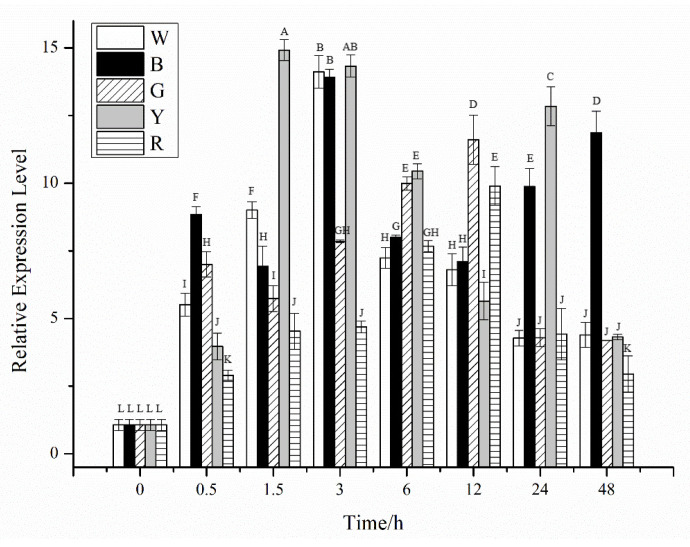
Relative expression of *CiPlant-CRY1* in different lights for different times. W: white light, B: blue light, G: green light, Y: yellow light, R: red light. Standard error bars are shown, n = 3. Different capital letters indicate significant differences between different treatments (*p* < 0.01).

The effects of light on the expression of *CiPlant-CRY1* are shown in Figure 5. In white light illumination, the expression level of *CiPlant-CRY1* reached the highest value of 14.111 at 3 h, but in blue light, the expression level fluctuated greatly, reaching a maximum value of 13.917 at 3 h. In green light, the expression level of *CiPlant-CRY1* generally showed a trend of first increasing and then decreasing, and reached a maximum level of 11.605 at 12 h, which was significantly higher than other experimental groups (*p* < 0.01). In yellow light, the expression level of *CiPlant-CRY1* fluctuated greatly, reaching the highest value of 14.916 at 1.5 h (*p* < 0.01). The expression level of *CiPlant-CRY1* in red light reached the highest value of 9.898 at 12 h.

**Figure 6:**
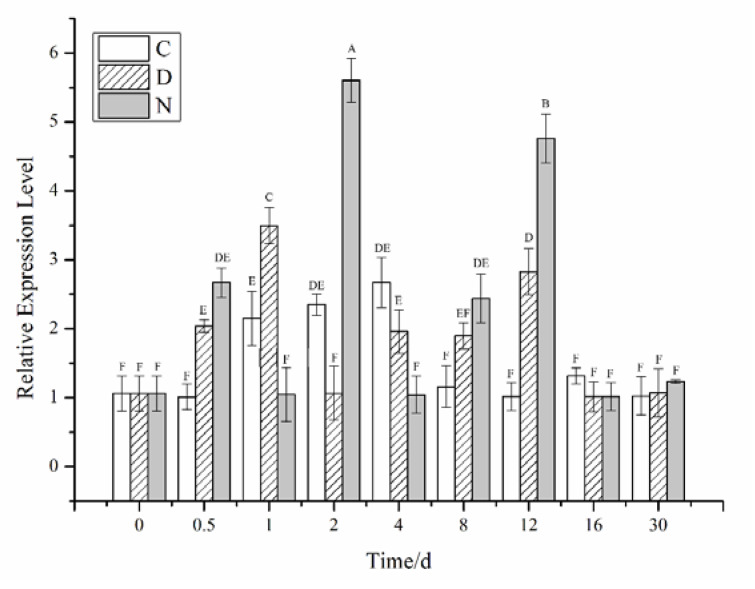
Relative expression of the *CiPlant-CRY1* in different photoperiods for different times. C: normal light (12 h light:12 h dark), D: polar day (continuous light), N: polar night (continuous dark). Standard error bars are shown, n *=* 3. Different capital letters indicate significant differences between different treatments (*p* < 0.01).

The relative expression of the *CiPlant-CRY1* in different photoperiods is shown in Figure 6, and the expression level of *CiPlant-CRY1* fluctuated greatly. In the normal light, the expression of *CiPlant-CRY1* reached a maximum value of 2.667 on the 4th day and then decreased. In the polar day conditions, the expression of *CiPlant-CRY1* reached a maximum value of 3.499 on the first day (*p* < 0.01). In the polar night conditions, the expression of *CiPlant-CRY1* was higher than other experimental groups at 0.5, 2, 8, 12, and 30 days, and reached the maximum value of 5.606 on the second day.

**Figure 7:**
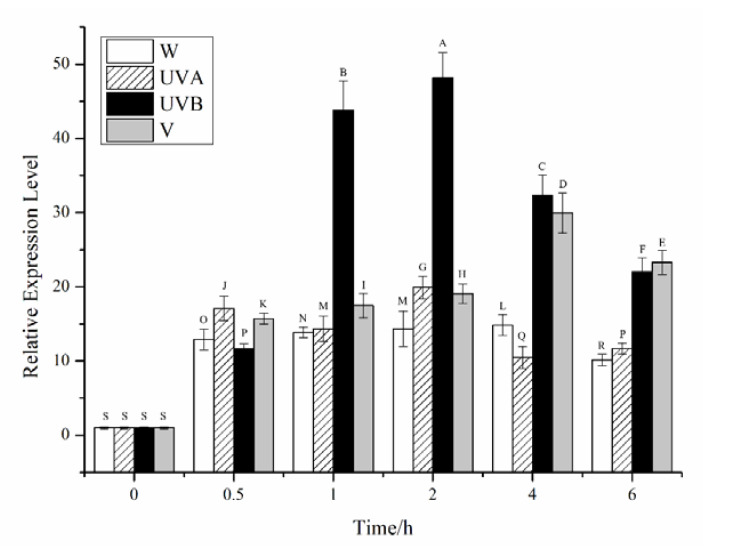
Relative expression of the *CiPlant-CRY1* in different ultraviolet lights for different times. W: white light, UVA: UVA irradiation, UVB: UVB irradiation, V: violet light. Standard error bars are shown, n *=* 3. Different capital letters indicate significant differences between different treatments (*p* < 0.01).

The relative expression of the *CiPlant-CRY1* in different ultraviolet lights is shown in Figure 7, and the expression levels of *CiPlant-CRY1* first increased, then decreased. The expression level of *CiPlant-CRY1* changed slightly in the normal light conditions. The expression levels of *CiPlant-CRY1* did not change much in UVA irradiation, but most of them were higher than that in normal light. UVB has the greatest effect on the expression of *CiPlant-CRY1*, which reached the highest value of 48.128 at 2 h (*p* < 0.01). In the violet light, the expression level of *CiPlant-CRY1* increased gradually within 4 h and reached a maximum value of 29.947.

### 2.3. Protein Expression

**Figure 8:**
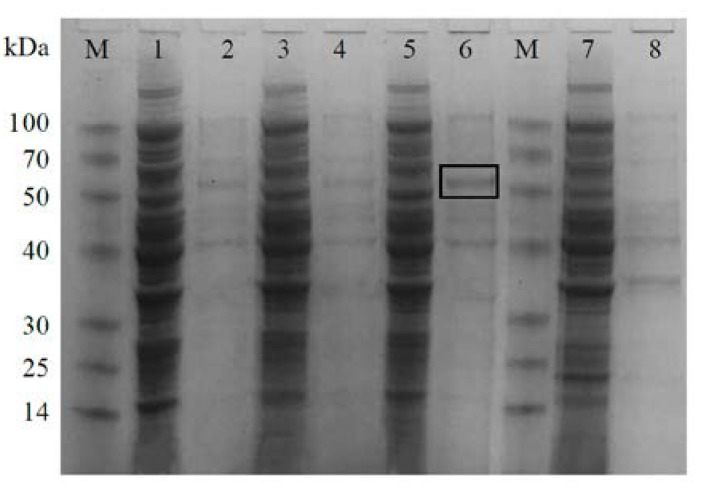
SDS-PAGE of the recombinant protein CiPlant-CRY1 induced by IPTG. M: Marker, 1: Uninduced CiPlant-CRY1 supernatant, 2: Uninduced CiPlant-CRY1 precipitate, 3: CiPlant-CRY1 supernatant induced by 0.2 mM IPTG, 4: CiPlant-CRY1 precipitate induced by 0.2 mM IPTG, 5: CiPlant-CRY1 supernatant induced by 0.5 mM IPTG, 6: CiPlant-CRY1 precipitate induced by 0.5 mM IPTG, 7: No-load bacterial supernatant induced by 0.5 mM IPTG, 8: No-load bacterial precipitate induced by 0.5 mM IPTG.

The recombinant strain Transetta (DE3)-pEASY^®^-Blunt E2-*CiPlant-CRY1* was induced to express by IPTG and subjected to SDS-PAGE (Figure 8). The DE3-containing bacteria were induced overnight at 16 °C with 0.5 mmol/L IPTG as a negative control. The result showed that there was an obvious protein band (in the box) in lane 6, which was located in the precipitate. Its relative molecular weight is consistent with the predicted protein relative molecular weight of 52.32 kDa, which can be judged as the CiPlant-CRY1 protein band. However, it also expressed in uninduced bacteria, and the reasons may be as follows: (a) this protein is well expressed and can be expressed without induction, (b) this protein is similar in size to a protein in the original strain, and the two protein bands run into a single band.

## 3. Discussion

Antarctic ice algae thrive in polar sea ice, which is an extremely important biosphere habitat on Earth. The sea ice environment is characterized by low temperature, no light or low light, and seasonal dynamic changes. During the seasonal changes in sea ice formation and melting, eukaryotic phytoplankton living in sea ice habitats experience dramatic changes in environmental factors such as light, temperature, and salinity, and a variety of microalgae are able to adapt to these changes and form distinct sea ice biomes, known as ice algae [12]. The unique physiological characteristics of ice algae are closely related to their ability to adapt to extreme environments of sea ice [13].

CRY has also been extensively studied in green algae, which appear to have a more complex set of CRYs than higher plants [7,8,14]. In addition to canonical plant CRY and CRY-DASH, green algae also have plant-like, animal-like, and cryptochrome/photolyase family (CPF) proteins [15]. At present, most of the studies on algal CRY focus on normal temperature algae, while research on low-temperature algal CRY is less common. We have reported the cloning and expression characteristics of the *CiCRY-DASH1* gene in *Chlamydomonas* sp. ICE-L [11]. In this paper, the *CiPlant-CRY1* gene of *Chlamydomonas* sp. ICE-L was characterized. The amino acid sequence of the *CiPlant-CRY1* gene only contained the conserved protein PHR domain shared by CRY, but no C-terminal extension functional domain. For plant CRYs, it has been shown that the C-terminal extension is partially destabilized upon illumination [16]. However, the motifs conserved in the C-terminal extension of land plants are not found in plant CRY from *Chlamydomonas reinhardtii* [7].

Plant CRY has recently been implicated in adaptations to temperature variation, including temperature compensation of the circadian clock [17]. Temperature is an essential factor affecting biological rhythm, and the biological clock has a temperature compensation effect, which means it can remain stable under certain temperature conditions [18,19]. The transcriptional regulation of the *CiPlant-CRY1* gene showed a certain change trend at −5 °C, 0 °C, and 5 °C, and the transcription level at −5 °C, 0 °C, and 5 °C was significantly higher than that at 10 °C, indicating that temperature can affect the expression of the *CiPlant-CRY1* gene. The suitable temperature for the growth of *Chlamydomonas* sp. ICE-L is 4–6 °C, and its biological rhythm will be destroyed when the temperature exceeds the suitable growth temperature.

Antarctic ice algae can survive and multiply in sea ice salt sacs. Dehydration, caused by the high brine salinities, is a major stressor for ice algae, which may experience salinities three times that of seawater. Conversely, when the ice melts, the released ice algae will be suddenly exposed to hyposaline conditions close to freshwater values [12]. The expression of the *CiPlant-CRY1* gene in *Chlamydomonas* sp. ICE-L was the highest at the optimal salinity of 32‰ and showed a certain regularity. In addition, its expression level under the salinity of 16‰ and 96‰ was significantly lower than that of the optimal growth salinity, indicating that salinity can affect the expression level of the *CiPlant-CRY1* gene.

In plants and algae, light serves both as the energy source for photosynthesis and a biological signal that triggers cellular responses via specific sensory photoreceptors [20]. To perceive fluctuations in light quality, quantity, and timing, plants and algae have evolved diverse photoreceptors including CRY [21]. Changes in the expression level of the CRY gene under different light qualities (white, blue, green, yellow, and red light) indicate that it can effectively respond to different light qualities, especially blue and yellow light. This reveals that *CiPlant-CRY1* may not only be categorized with respect to a potential role as a blue light receptor, but yellow light activation of *CiPlant-CRY1* needs to be considered as well in the design of future experiments [22]. Moreover, *CiPlant-CRY1* can maintain a high level of expression through the treatment of blue light, which indicates that CiPlant-CRY1 is involved in the formation of light morphology, regulation of pigment biosynthesis, light capture complex synthesis, cell cycle, etc., as a blue light receptor [8].

Antarctica represents an extreme light regime with an annual light cycle including periods of polar day and polar night. The expression of the *CiPlant-CRY1* gene is highly responsive to long-term light and dark treatments, and shows a certain circadian rhythm, indicating that its expression is regulated by circadian rhythm [19]. Light/dark cycles entrain the *Chlamydomonas* circadian clock, and *CiPlant-CRY1* belongs to the biological clock component [23,24]. Therefore, analyzing the circadian expression of *CiPlant-CRY1* in long-term light and dark environments can help to understand the mechanisms of plant circadian regulation.

CRYs are photolyase (PHO)-like flavoproteins and together constitute a CPF that has been found in all evolutionary lineages [4,25]. The CPF includes structurally similar proteins that function differently and use near-ultraviolet (UV)/blue light photons as an energy source to mediate either DNA repair or perception and signal transduction of external light signals [22,26,27]. The ozone hole over Antarctica may produce massive amounts of UV radiation. The expression level of *CiPlant-CRY1* was most affected by UVB irradiation. CRY blue light signaling shows some similarities to UVR8 UV-B signaling [28]. We speculate that the *CiPlant-CRY1* gene is sensitive to UVB and may be related to the adaptation of Antarctic ice algae to strong ultraviolet radiation environments.

## 4. Materials and Methods

### 4.1. Algal Culture

The Antarctic ice alga *Chlamydomonas* sp. ICE-L was isolated from the floating ice near the Zhongshan Research Station of Antarctica (69° S, 77° E) [29]. Cultures were routinely grown in autoclaved Provasoli seawater medium [30] at a light density of 40 μmol m^−2^s^−1^ with a diurnal cycle of 12 h light and 12 h dark at a temperature of 5 ± 1 °C.

### 4.2. RNA Extraction and cDNA Synthesis

Total RNA was extracted using TRIzol reagent (TransGen Biotech, Beijing, China) according to the manufacturer’s instructions. RNA integrity was determined by electrophoresing the sample on a 1% agarose gel, while RNA quantification and purity were determined using a NanoDrop 2000 spectrophotometer (Thermo Fisher Scientific, Wilmington, DE, USA). cDNA synthesis was performed using the PrimeScript™ II 1st Strand cDNA Synthesis Kit (Takara, Dalian, China).

### 4.3. Cloning and Sequencing of CRY Complete ORF

Specific primers (Table 1) were designed according to the potential full-length ORF of *CiPlant-CRY1* obtained from the *Chlamydomonas* sp. ICE-L transcriptome (BioSample ID: SAMN15165724), and the cDNA fragments related to the *CiPlant-CRY1* gene were amplified by PCR. PCR amplification was performed as follows: 95 °C for 2 min; 40 cycles of 95 °C for 30 s, 55 °C for 15 s, 72 °C for 20 s; and 5 min at 72 °C. The PCR products were purified using the EasyPure^®^ RNA Purification Kit (TransGen Biotech, Beijing, China). Then, the purified PCR fragments were ligated into the pEASY^®^-Blunt cloning vector (TransGen Biotech, Beijing, China) and transformed into the Trans1-T1 Phage Resistant Chemically Competent Cell (TransGen Biotech, Beijing, China). Three randomly selected clones were identified as positive clones from Amp+/LB plates and then sent to Sangon Biotech (Shanghai, China) for DNA sequencing.

### 4.4. Bioinformatic Analysis

BLAST was used to test the homology of *Ciplant-CRY1* genes (http://blast.ncbi.nlm.nih.gov/Blast.cgi (accessed on 7 April 2021)); Open reading frame (ORF) analysis was performed with the NCBI ORF Finder (http://www.ncbi.nlm.nih.gov/gorf/gorf.html (accessed on 7 April 2021)). Multiple sequence alignment was performed using the Clustal X program and subsequent analysis was performed by the BioEdit program [31]. The gene sequence of *CiPlant-CRY1* was translated into amino acid sequences (http://web.expasy.org/translate/ (accessed on 7 April 2021)), and the amino acid homology analysis was performed by DNAman. The physicochemical properties, secondary structure, transmembrane domain, subcellular localization, and tertiary structure of the protein were analyzed using the following software and website: ExPASy (https://web.expasy.org/protparam/ (accessed on 8 April 2021)); predictProtein (http://www.predictprotein.org (accessed on 8 April 2021)); TMPRED (http://embnet.vital-it.ch/software/TMPRED_form.html (accessed on 8 April 2021)); WoLF PSORT (http://www.genscript.com/wolf-psort.html (accessed on 8 April 2021)). Mega 6.06 software was used to generate a phylogenetic tree based on the neighbor-joining method and evaluated by a bootstrap analysis with 1000 replicates [32].

### 4.5. Analysis of CiPlant-CRY1 mRNA Expression by Quantitative Real-Time PCR (qRT-PCR)

Logarithmic phase algal cells grown in normal culture were subjected to different stress conditions to study the relative expression of the *CiPlant-CRY1* gene. In the temperature stress treatment, cells were exposed to a gradient of temperatures: −5, 0, 5 (normal temperature), and 10 °C, and cells were harvested at 0, 2, 4, 6, 8, 12, 24, and 48 h. Salinity stress was created with Provasoli medium containing NaCl at final concentrations of 16‰ (0.5-fold), 32‰ (normal sea water), 64‰ (2-fold), and 96‰ (3-fold) for 0, 2, 4, 6, 8, 12, 24, and 48 h. In the light quality treatment, cells were cultured in white (normal), blue (440–460 nm), green (500–560 nm), yellow (560–590 nm), and (620–750 nm) red light with 5 W power lamps (Qihui Lighting Company, Shenzhen, China), and the lamps were 20 cm away from cells for 0, 0.5, 1.5, 3, 6, 12, 24, 48 h. Under photoperiod stress, cells were cultured in normal conditions (12 h light:12 h dark), and continuous light (polar day) and dark (polar night) treatments, respectively, for 0, 0.5, 1, 2, 4, 8, 12, 16, 30 d. In the ultraviolet stress treatments, cells were cultured in normal conditions (white light), UVA (320–400 nm), UVB (280–320 nm, 95 μW cm^−2^), and violet light (400–440 nm) for 0, 0.5, 1, 2, 4, 6 h with 18 W power lamps (Nanjing Huaqiang Electronic Company, Nanjing, China) 20 cm away from cells. In all treatment groups, only single factors such as temperature, salinity, light quality, photoperiod, and ultraviolet were changed, others were the same as normal culture conditions, and there were three replicates for each treatment.

*CiPlant-CRY1*-specific primers (Table 1) were designed using Primer Premier 5.0 software (PREMIER Biosoft International, San Francisco, CA, USA). The housekeeping gene, 18S ribosomal RNA, of *Chlamydomonas* sp. ICE-L was used as an internal control. qRT-PCR was performed with a LightCycle^®^ 96 Instrument (Roche Diagnostics, Indianapolis, IN, USA) and TransStart^®^ Green qPCR SuperMix (TransGen Biotech, Beijing, China). The qRT-PCR amplification programs included an initial denaturation step of 94 °C for 30 s; followed by 42 cycles of 94 °C for 5 s, 58 °C for 15 s, and 72 °C for 10 s. The data were further analyzed by the comparative Ct (2^−ΔΔCT^) method [33]. The LSD test and average ± SD of each experiment were calculated and analyzed using IBM SPSS Statistics 22.0 data analysis software (IBM Corp., Armonk, NY, USA).

### 4.6. Protein Expression

The PCR product was ligated into a pEASY^®^-Blunt E2 Expression Vector (TransGen Biotech, Beijing, China), which was then transformed into a Trans1-T1 Phage Resistant Chemically Competent Cell (TransGen Biotech, Beijing, China). Subsequently, the correctness of the *CiPlant-CRY1* sequence and ligation direction was verified. The plasmid was extracted from the bacterial solution with correct expression direction and then transformed into a Transetta (DE3) Chemically Competent Cell (TransGen Biotech, Beijing, China). Finally, isopropanol-β-D-thiogalactoside (IPTG) was used to induce the expression of the CiPlant-CRY1 protein in the bacterial solution with the correct expression direction.

## 5. Conclusions

In this study, a novel plant type CRY gene *CiPlant-CRY1* was first cloned and analyzed from Antarctic ice alga *Chlamydomonas* sp. ICE-L. qRT-PCR analysis revealed that *CiPlant-CRY1* is an important photoreceptor gene in response to blue or yellow light. Its expression is regulated by circadian rhythm and may be related to the adaptation of ice algae to strong ultraviolet radiation environments. The heterologous expression of the recombinant *CiPlant-CRY1* gene laid the foundation for further functional studies. This study provides a novel view of the mechanisms by which Antarctic ice algal CRY photoreceptors adapt to extreme sea ice environments.

## Figures and Tables

**Table 1:** Primers used in this study.

Role	Name	Sequence (5′–3′)
cDNA	F1	ATGCGCGTTGTGGCCGC
	R1	CCACATCTTCTGTCGTTTCTGGGG
qRT-PCR	F2	GTCGAAGGATGTGCCAAGGG
	R2	GGTGCCATCACCAGTTCCTG
18S rRNA	F4	ATGGAATAACACGATAGGACTCTGG
	R4	ACCTCTGACAATGAAATACGAATGC

## Data Availability

Not applicable.
